# Lessons learned from the hospital to home community care program in Singapore and the supporting AI multiple readmissions prediction model

**DOI:** 10.1002/hcs2.44

**Published:** 2023-05-10

**Authors:** John Abisheganaden, Kheng Hock Lee, Lian Leng Low, Eugene Shum, Han Leong Goh, Christine Gia Lee Ang, Andy Wee An Ta, Steven M. Miller

**Affiliations:** ^1^ Department of Respiratory and Critical Care Medicine, Tan Tock Seng Hospital National Healthcare Group Singapore Singapore; ^2^ National Working Group for the Hospital to Home Program Singapore Singapore; ^3^ Department of Family Medicine and Continuing Care, Singapore General Hospital, SingHealth Group Singapore Singapore; ^4^ SingHealth Community Hospitals, SingHealth Group Singapore Singapore; ^5^ Population Health and Integrated Care Office, SingHealth Group Singapore Singapore; ^6^ Office of Community Development Changi General Hospital, SingHealth Group Singapore Singapore; ^7^ Data Analytics and AI Department, Integrated Health Information Systems Singapore Singapore; ^8^ School of Computing and Information Systems Singapore Management University Singapore Singapore

**Keywords:** hospital to home community care, hospital to home lessons learned, transitional care, integrated care, multiple readmissions AI prediction model, machine learning in healthcare, healthcare technology

## Abstract

In a prior practice and policy article published in Healthcare Science, we introduced the deployed application of an artificial intelligence (AI) model to predict longer‐term inpatient readmissions to guide community care interventions for patients with complex conditions in the context of Singapore's Hospital to Home (H2H) program that has been operating since 2017. In this follow on practice and policy article, we further elaborate on Singapore's H2H program and care model, and its supporting AI model for multiple readmission prediction, in the following ways: (1) by providing updates on the AI and supporting information systems, (2) by reporting on customer engagement and related service delivery outcomes including staff‐related time savings and patient benefits in terms of bed days saved, (3) by sharing lessons learned with respect to (i) analytics challenges encountered due to the high degree of heterogeneity and resulting variability of the data set associated with the population of program participants, (ii) balancing competing needs for simpler and stable predictive models versus continuing to further enhance models and add yet more predictive variables, and (iii) the complications of continuing to make model changes when the AI part of the system is highly interlinked with supporting clinical information systems, (4) by highlighting how this H2H effort supported broader Covid‐19 response efforts across Singapore's public healthcare system, and finally (5) by commenting on how the experiences and related capabilities acquired from running this H2H program and related community care model and supporting AI prediction model are expected to contribute to the next wave of Singapore's public healthcare efforts from 2023 onwards. For the convenience of the reader, some content that introduces the H2H program and the multiple readmissions AI prediction model that previously appeared in the prior Healthcare Science publication is repeated at the beginning of this article.

## Graphical abstract

This article elaborates on Singapore's Hospital to Home program and care model, and its supporting AI model for multiple readmission prediction, in the following ways: by providing updates on supporting information systems, by reporting on customer engagement and related service delivery outcomes, by sharing three categories of lessons learned, by highlighting how this effort supported broader Covid‐19 response efforts, and by commenting on how the experiences from running this program since 2017 are expected to contribute to the next wave of Singapore's public healthcare efforts from 2023 onwards.

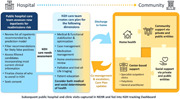

**Abbreviations**
AI: artificial intelligence
BRAIN: Business Research Analytics Insight Network platform
H2H: Hospital to Home program
NEHR: National Electronic Health Records system

## 1 INTRODUCTION

Elderly patients, especially those with multiple chronic ailments and complex care needs, often encounter challenges with taking care of themselves after being discharged from a multiday hospital stay, and this leads to a high number of related readmissions over the 12–24 months after discharge [1, 2]. To reduce these types of readmissions, in April 2017, the Singapore public healthcare system implemented the Hospital to Home (H2H) National program to provide a nationwide community care framework for supporting this special subset of patients—which included many frailer and elderly patients with higher levels of medical and social support needs—after their discharge from public hospitals. A major challenge was to determine which of all the new inpatients admitted each day should be enrolled into the H2H program. To meet this challenge, an AI prediction model trained on health record and demographic data was deployed to do the initial stage of patient screening for program enrollment. Those identified by the model to be at high risk for multiple readmissions were further considered in a second stage, clinical judgement‐based evaluation. This much faster AI‐guided identification of the initial pool of candidates gave the care team more time to work and engage with the targeted patients during their hospital stay to prepare them for what to do after discharge. The H2H program aimed to provide a safe and timely transition from acute hospital inpatient stay back to the home and community environment for these types of patients at risk for multiple readmissions. Postdischarge community care practices used by the H2H program substantially benefited from the experiences of prior local community care pilot efforts [3, 4]*.

## 2 THE SUPPORTING AI PREDICTION MODEL AND THE H2H PROCESS FLOW

**Figure 1:**
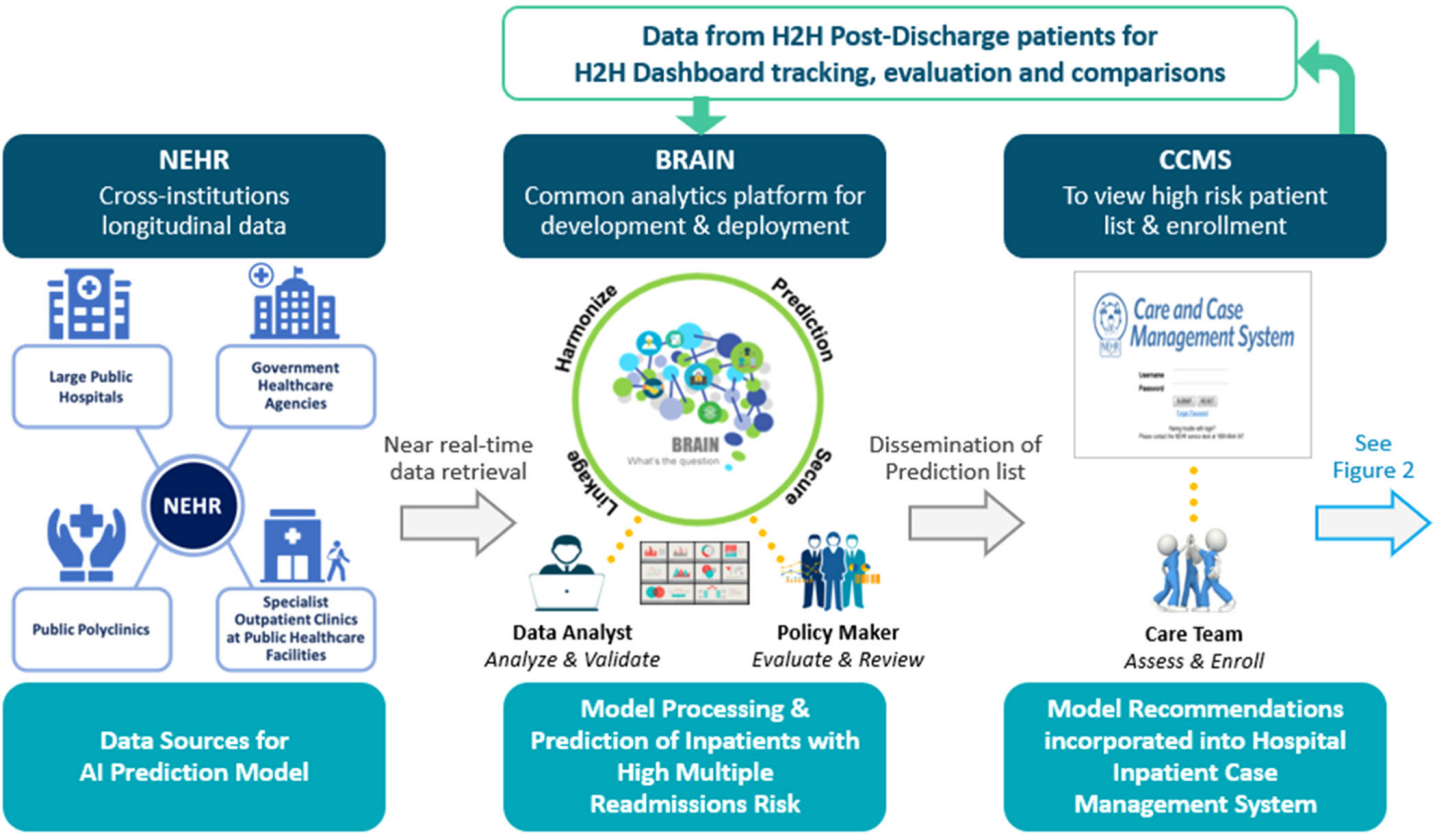
The three major information systems required for generating and using the Multiple Readmissions AI Prediction Model to support the Hospital to Home (H2H) patient selection process. AI, artificial intelligence.

A supporting artificial intelligence (AI)‐based multiple admissions prediction model was used to provide a starting point for identifying which subset of patients recently admitted to any public hospital should be placed in the H2H program because of the combination of their complex postdischarge support needs and their high risk of returning as an inpatient, not just once but likely several times over the next 12 months. As shown in Figure 1, all input data for the prediction model originated from the National Electronic Health Record (NEHR) system. All data flowed into the Business Research Analytics Insight Network (BRAIN) platform, a centralized business intelligence, analytics, and AI processing platform serving a wide range of data analytics needs across Singapore's public healthcare system. Within BRAIN, a scheduler system triggered the daily running of the multiple readmissions prediction model for all patients recently admitted into public hospitals and generated a patient risk score for readmissions.

The output of the prediction model was an identification of those patients admitted within the past 24 h to a particular public hospital assessed as having a high risk of multiple readmissions over the next 12 months. This output was incorporated into the hospital's Care and Case Management System (CCMS) which provided a standardized view of each patient currently admitted into that hospital and included their admissions details, demographics, and past medical intervention history within the public health system.

Input data for the AI prediction model included over one thousand indicators segmented into three primary categories: sociodemographic characteristics, past hospital utilization information, and information on past medical conditions. Examples of input data indicators included patient age and demographics, number of nonelective inpatient admissions and total length of hospital stays in the past 2 years, and total number of specialist outpatient visits and emergency department visits in the past year.

As different types of machine learning algorithms have their respective advantages and drawbacks, we developed and tested the following types of machine learning‐based predictive models: logistic regression, lasso, random forest and gradient boosting machine, and compared the results. To derive and internally validate the prediction models, the cohort was randomly split into a training data set (80%) and a test (validation) data set (20%). To avoid overfitting problem, 10‐fold cross validation was used on the training data set to determine the tuning parameters.

To determine the performance of the prediction model, the following metrics were used: Sensitivity, Positive Predictive Value (PPV), and Area‐Under‐Curve (AUC). After consultation with the H2H clinical committee, the PPV of the model was set at 70% to capture more potential upstream patient participants, as setting a higher PPV would have resulted in only very high‐risk patients being identified. Based on PPV 0.7, the Gradient Boosting Machine was the best‐performing machine learning algorithm, giving an AUC of 0.79 and sensitivity of 39%.

An additional consideration in model choice was that model explainability was considered an essential requirement which is why deep learning systems were not used. The gradient boosting algorithm has a high degree of interpretability because it is possible to show the relative influence of each variable on model output and clinician subject matter experts are able to check if these relative influences are in concordance with their clinical judgment. Given our overall considerations encompassing both overall predictive performance and interpretability, the gradient boosting algorithm was finally selected for deployment. Elaboration on how the clinical context and workflow influenced model design and usage, and on technical details of model design are given in [5] and [6].

**Figure 2:**
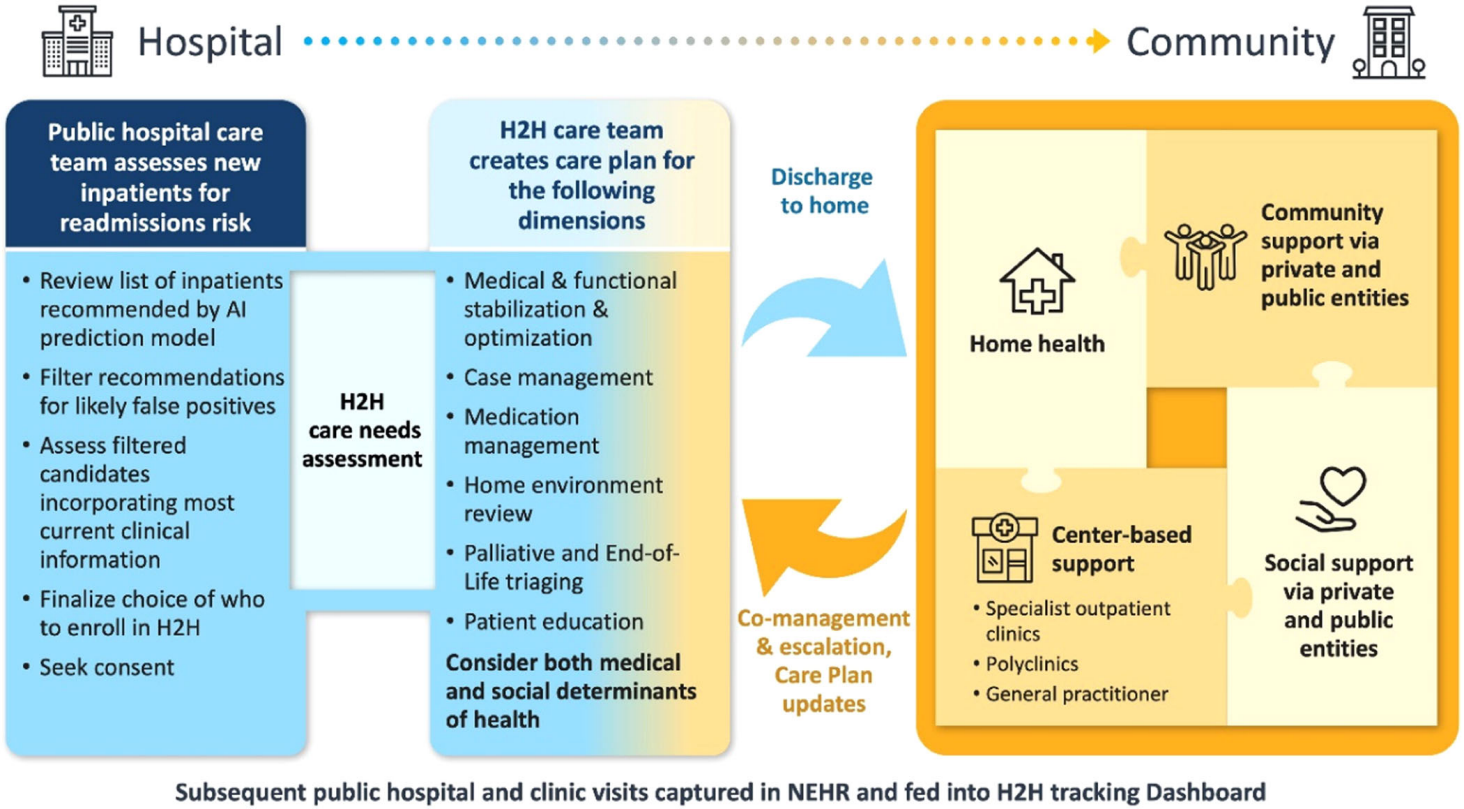
The Hospital‐to‐Home (H2H) Care Model. Using the recommendations of the AI prediction model as a starting point for identifying candidates for the H2H program, clinical staff continue to evaluate and select patients to enroll, prepare a care plan, and educate enrolled patients for what to do after discharge back to home and the community. The community‐based integrated care team works with an ecosystem of community partners to support the discharged H2H program participants to reduce risk of subsequent hospital readmissions. AI, artificial intelligence.

As shown in Figure 2, hospital staff used the predictive model as a sieve to do the first round of filtering for identification of inpatients for inclusion in the H2H program; they then did a second round of clinical judgment, needs‐based vetting, and consideration to finally select which inpatients to invite into the program. Inpatients who agreed to enroll in the H2H program received program‐specific counseling and education during the remainder of their hospital stay.

After discharge, they received care and support through home visits and phone follow‐ups by an interdisciplinary team of healthcare professionals (doctors, nurses, allied health professionals, social workers, and community care coordinators). To the extent practically possible, H2H care teams would monitor patients discharged back to their home and community settings and address risk factors for clinical deterioration such as noncompliance with medications, poor health literacy, and polypharmacy (regular use of five or more medications). The care teams did this through directly communicating with discharged patients via community care interactions and by tracking through the dashboard data indicating subsequent visits to public health facilities (neighborhood clinics, hospital outpatient departments, or inpatient readmissions).

Each large public hospital participating in the H2H program made had their own somewhat customized version of the H2H care model shown in Figure 2. Each hospital made substantial operational adjustments over the 2017 to 2019 period as they learned how to integrate their respective within‐hospital H2H workflows (the right side of Figure 1 and left side of Figure 2) with the workflows of their other hospital processes, and also as they learned to partner more effectively with their respective network of community‐based healthcare and social support entities (the right side of Figure 2). It took several years of relationship‐building effort to establish high degrees of trust between the upstream parts of the H2H process (all of Figure 1 and the left of Figure 2) and the downstream parts (the right half of Figure 2).

## 3 UPDATES TO THE AI APPLICATION AND SUPPORTING INFORMATION SYSTEM SINCE INITIAL DEPLOYMENT

With the start of the H2H program in 2017, the supporting AI model that was concurrently deployed was the very first time that an AI prediction model was used across all of Singapore's public hospitals as part of their daily work related to patient assessments. It was also the first public healthcare demonstration of the viability of using routine data found in the NEHR system to build an AI‐based prediction tool to support patient care [6]. In terms of comparisons to international healthcare practices at that time, it differed from the more common clinical practice approach of predicting inpatient readmission only within the next 30‐day period, as the focus of the H2H program was to ensure longer‐term continued care and support for patients with complex care needs after discharge from hospital.

From 2018 through 2022, H2H program managers and ministry‐level national health policymakers decided to keep the AI prediction model essentially unchanged (aside from adjustments for model drift) to give clinicians who were using the model outputs in the hospital wards a stable and familiar tool to work with. Important aspects of the supporting technology that did change during this time were as follows:

a. *Substantial improvements to the underlying* BRAIN platform *for handling all required data management and analytics processing*.

BRAIN is a centralized business intelligence, analytics, and AI processing platform built by Integrated Health Information Systems (IHiS, Singapore's national healthtech agency) [7] to serve a wide range of data analytics needs across Singapore's public healthcare system, including the multiple readmissions prediction model. A major improvement to BRAIN implemented during this time was the establishment of machine learning operations processes and support tools (MLOps) which made it possible to detect and respond to data drifts.

b. *The addition of an H2H tracking, reporting, and evaluation dashboard*.

This dashboard was used by program‐level analysts and decision‐makers within the hospitals and at the ministry level for tracking and evaluating H2H efforts and for future planning. Users could do many types of reporting and exploration. An especially important feature of the dashboard was that outcomes of different subgroups could be tracked and compared over time. For example, the dashboard could be used to compare the status and outcomes of three different groups of elderly patients:

- Those with various co‐morbidities who entered the H2H program.
- Those recommended by the model but who were not enrolled in the program, either because they were filtered out by clinical staff after the clinical assessment phase, or because the patients declined to participate.
- Those who were not recommended by the multiple readmissions prediction model.

This enabled the matching of suitable controls for comparative studies. These comparisons were especially helpful given that randomized control trials were extremely challenging, if not outright impractical, to implement under these circumstances and at this scale. The dashboard also allowed each participating public hospital to view H2H program outcomes and trends at other hospitals. Thus, each hospital could benchmark themselves. This facilitated iterative improvements of the entire end‐to‐end H2H process and care model (while keeping the AI prediction model essentially unchanged) through sharing of best practices within hospital teams and across participating hospitals and their respective community support ecosystems.

As shown at the top of Figure 1, the H2H dashboard integrated data from BRAIN and the CCMS and from several other systems as well and provided a cohesive view of key outcome indicators, making it possible to identify trends and areas for program delivery improvements. Developing and deploying this dashboard required substantial collaborative efforts to design and test functionality and usability. Clinicians, healthcare management and policy analysts, and IT and data analytics specialists worked in tandem using an agile, iterative process to validate and improve the dashboard's performance.

c. *The addition of a supplemental prediction model of readmission risk within the next 30 days*.

After the clinical staff viewed the list of inpatients recommended by the 12‐month horizon multiple readmissions prediction model, they then looked at a separate 30‐day horizon readmissions prediction model and used it as part of their second round of clinical judgment‐based vetting and consideration for finalizing the choice of which patients to invite into the H2H program. There were efforts to evaluate other stand‐alone supplemental AI‐based predictions models that were hypothesized to be relevant to the 12‐month horizon multiple readmissions prediction. Other supporting models that were created and tested included prediction of likelihood of an accidental fall after discharge, and a more targeted prediction of readmission over the next 12 months specifically for those patients who were already living in nursing homes. While each of these supplementary models had useful predictive value, H2H program decision‐makers chose not to proceed with them. Clinicians felt that the addition of several additional scores was distracting from the effort of making a holistic clinical assessment of who to invite into the H2H program based on an individual patient's care needs.

## 4 CUSTOMER ENGAGEMENT AND RELATED SERVICE DELIVERY OUTCOMES

Without using the AI multiple readmissions prediction model as a screening tool, nurses in each public hospital screening for H2H program candidates would have needed to spend about half of every day manually reviewing the entire patient list and going round the wards to assess the situation with the newly admitted patients and their care team to identify those at higher risk of multiple readmissions. This would have been too time intensive and would have made it impractical to identify H2H program candidates on a daily basis within every ward of every public hospital.

Only between 10% and 15% of all newly admitted patients were identified by the prediction model as meeting the designated threshold for elevated risk of multiple readmissions over the next 12 months. Nurses, doctors, and other clinicians involved in H2H program selection could therefore focus on this identified subset as a starting point, and then more quickly proceed to the second round of clinical judgment‐based vetting to make the final candidate selection, thus avoiding the need to sort through the other 85%–90% of the recently admitted patients.

Given there were well over 450,000 inpatient admissions per year to Singapore public hospitals (with around 40% of this total being people 65 and older) [8]†, the use of the AI‐based prediction model to do the first round of identification of relevant H2H candidates reduced the average daily vetting workload from over 1200 patients per day (if all inpatients across all public hospitals had to be reviewed) down to an average between 123 and 183 per day (10%–15% of nationwide average daily admissions), making the required daily vetting workload within each public hospital a manageable task. In addition, the faster selection process facilitated by the AI prediction model enabled the H2H multidisciplinary care team to have more engagement time during the current hospital stay with those patients who did enter the program. This additional engagement time turned out to be crucial for preparing the enrolled patients and their family members for what to do postdischarge.

Without the support of the AI prediction model, we approximate it would have taken the nursing teams handling the H2H workload three times longer to do the daily first‐step identification of program candidates. That would have delayed the initiation of the second‐step clinical vetting for finalizing which patients to enroll by 2 to 3 working days, which in turn would have reduce the amount of time within the current inpatient stay that doctors and other care team members had to educate the patient and their family on what to do postdischarge. Clinicians in the H2H program appreciated the reduction in their workload resulting from AI‐supported screening. This time savings allowed doctors, nurses, and other members of the care team to focus their effort on patients—both the current inpatients who are being enrolled into the program as well as those who were already discharged and part of the community care network.

An unexpected peer engagement benefit that organically emerged from the deployment of the AI model was the formation of a cross‐hospital community of practice user groups. Comprised of H2H program leaders, the group meet regularly since the start of model validation in 2017 through the end of 2022 to share experiences using the model and suggestions for improving model usage in the context of hospital clinical practice and community care. In addition to helping the AI model deployment and overall H2H program, this informal sharing among peers led to other important cross‐hospital informal exchanges and relationship building.

While the AI prediction model was an essential support tool for the initial step of the H2H candidate selection process, its usage does not inform us about the downstream benefits of getting inpatients enrolled into the H2H program. We used the net change in inpatient bed days before and after enrollment as the measure for making this type of impact assessment because this measure was incorporated into the dashboard and could be viewed by the national level H2H working group as well as by the H2H staff in the participating hospitals. While other measures such as before and after net changes in readmissions or emergency room visits would also have been good impact measures, these other measures were not incorporated into the dashboard and were therefore not available to us as a means of comparison and impact evaluation.

The average number of bed days saved across three of our major hospitals represented by our clinician co‐authors ranged from 3 to 7 days per H2H patient enrolled in the 180 days postprogram enrollment for the period between April 2019 and April 2021. For this measure, we compared patients who were enrolled into the H2H program versus a group who were not enrolled but who had a similar age and Charlson Comorbidity Index score [9, 10]. For both groups, we compared the cumulative measure of bed days 180 days post an “index” admission versus the cumulative measures 180 days before the index admission‡. As clinicians, we view the bed days saved as added value to the patients (via hospital inpatient stay reduction or avoidance) as well as to the hospital (via more available bed capacity).

In summary, the AI prediction model provided the H2H program staff with a time‐efficient support tool for the first step of the candidate identification and selection process. Getting people enrolled in the H2H program has resulted in health outcome benefits as demonstrated by the reductions in inpatient bed days as reported above.

## 5 LESSONS LEARNED

### 5.1 Related to engaging and supporting H2H program participants

Superficially, it might seem like the patients who entered the H2H program were a homogenous group in that most were elderly, and many were frail. Clinically, it is just the opposite; the patients considered for and finally enrolled into H2H were a highly heterogeneous group in that most had multiple chronic diseases [11]. Each patient's medical condition and associated presentation of symptoms was quite distinct, complex in their own way, and was associated with different risk levels for postdischarge complications that could require rehospitalization. The situation was further compounded by the wide variation in social support and conditions that influence social determinants of health across the H2H participant population. The heterogeneity of patients being considered and enrolled was a key reason why the two‐step selection process for inclusion in the program was adopted with the first step using the AI prediction model as a sieve to identify the pool of relevant patients and the second step for further filtering and evaluating potential participants based on a clinical assessment of conditions and care needs.

The H2H program included sizable numbers of inpatients who were rejected by or not included in mainstream disease‐specific trials, support programs, or special interventions because of their highly complex multimorbidity conditions. The complexity and nonheterogeneous nature of H2H patients made comparisons with controls and evaluation particularly challenging, even with the data from the dashboard. We found it was especially difficult to prove or show success with cohorts of patients in the H2H program as compared with patient cohorts enrolled in more disease‐specific programs focused on one main chronic condition [12]§.

What we do know for sure is that this special subpopulation of people enrolled in the H2H program comprised 10%–15% of the total public hospital inpatient population and consumed a disproportionately high percentage of public healthcare resources and associated costs, with some international estimates at 50% of the total cost [13, 14] and even higher [15]. In this day and age when government decision‐makers and senior healthcare policymakers increasingly expect analytics to provide clear‐cut evaluation data and unambiguous insights, we are humbled by the realities of working with the H2H population where the complex and dynamic health and social conditions of each patient resulted in cohort evaluation data that was inherently noisy and highly variable, and was not easily amenable to straightforward evaluations and comparisons.

While AI methods were tremendously helpful in the front‐end of the H2H process to recommend who to enroll into the program, we did not attempt to use other appropriate types of AI and related statistical methods to support the evaluation of outcomes for individuals or cohorts after enrollment. At the outset and during the earlier years of the H2H program, we viewed the effort of building AI models to support postenrollment assessment too difficult and time‐consuming to undertake due to the complex factors discussed above and also due to our focus on meeting the challenges of implementing the end‐to‐end workflows shown in both Figures 1 and 2. In the future, as more data on H2H participant outcomes is accumulated over longer time periods, we recommend that special studies be designed, reviewed, approved, and launched that use the available data and construct additional evaluation measures beyond the one metric of net change in inpatient bed days before and after enrollment that was available to us through the H2H program dashboard.

### 5.2 Related to the use of AI predictive models to support the H2H program

Rather than adding separate, additional specialized risk prediction models to supplement the original multiple readmissions model, a “multi‐model” ensemble framework needs to be devised that can reliably combine the results of multiple prediction models into one holistic readmissions‐related risk score in a way that clinicians can easily understand and use. Clinicians want and need more than the prediction of which inpatients are at high risk of readmission over either the near‐term 30‐day horizon or the longer‐term 12‐month horizon. They also need to know why the patient is flagged as being high‐risk. It would be even better if the prediction tool could recommend an appropriate intervention or next course of action, especially for situations when the care need is not immediately apparent to the clinician.

This creates challenges we need to carefully navigate. Clinicians want a stable prediction model to work with in order to become familiar with and calibrated to the model's capabilities as well as limitations. As such, we do not want to see the model changing too frequently. Of course, over time, clinicians also want to see improvements made to the model. When introducing a new variable, it is easier to test it and to interpret the meaning of its outputs in a stand‐alone fashion. This leads to the issue mentioned above that clinicians do not want to have to cope with the added complexity of using multiple assessment models. Often the prediction power of a new variable is based on how it interacts with many of the already existing variables in the existing model, so testing a new variable in a stand‐alone model may not show the full advantages of including that new variable into the multivariable model. Yet, continuing to add new variables to the existing multivariable model makes it harder for the clinicians as well as the supporting data scientists to understand how the behavior of the prior, familiar version of the model has changed. In summary, once an AI prediction model is introduced into a hospital's workflow for recommending patient suitability for a specific care program and proves to be useful and satisfactory, the subsequent process of making changes to enhance the model needs to be a very carefully controlled, thoroughly tested, explainable and gradual process.

Basing the initial multiple readmissions model on data contained within the NEHR system was a natural starting point and proved to be a good strategy for a workable and scalable solution. However, we strongly suspect this type of prediction model would benefit from the addition of variables related to the person's ability to perform activities of daily living, socioeconomic variables, and several psychological variables. These types of data are not conveniently consolidated in one place, so exploring how to make use of these other types of variables will require a careful trade‐off analysis between additional predictive power and practical availability.

We also learned that our ability to correctly predict who is likely to require multiple readmissions over the 12‐month future horizon using the combination of the AI model and clinical assessment sometimes goes beyond our ability to intervene and influence the outcome. After the initial 3 years of H2H operation, we realized that certain types of H2H patients ended up requiring multiple readmissions despite the fact that we correctly identified this risk, created a customized care plan, and provided them with postdischarge home and community‐based support. This was the situation for patients who had serious mental health issues (including substance abuse, alcohol‐dependence, personality disorders), patients approaching the end of life, and patients who totally ignored the care plan or had very low levels of adherence. After observing these results, several of the public hospitals decided to make a “course‐correction” and refocused on enrolling patients who would more likely benefit from the H2H program. For the hospitals that made this course correction, we did not change the AI prediction model as the model was working as intended. Rather, we adjusted our second step of clinical assessment, screening, and selection criteria.

### 5.3 Related to the overall use of information systems to support the H2H program

While the AI‐based multiple readmissions prediction model was a critical enabler of the overall H2H program, it was just one part—a relatively small though critical part—of the overall set of information systems required to run the H2H effort. The ability to track the results and analyze the impacts of the H2H program was enabled through the H2H dashboard, which is a “conventional” type of business analytics reporting system. Both the AI multiple readmission prediction model and the H2H dashboard required the enabling support of the central BRAIN platform. BRAIN has been supporting many basic yet essential data management and business analytics functions, as well as the AI‐related model development and production usage. In addition, output from the AI prediction model was integrated into the Care and Case Management information systems used by the hospital staff to manage their inpatients as well as into other information systems used by hospital staff (Figure 1).

It is important for those at both the hospital level as well as at the government level involved with shaping, executing, and overseeing healthcare programs such as H2H that use AI methods as a critical enabler of part of the workflow to appreciate the entire set of information systems required to deploy and use an “AI model” in a production setting. The complex interdependencies across these various information systems is another reason why a more gradual and very thoughtfully considered approach to changing the deployed AI model is a sound approach in the context of highly interdependent clinical care settings and supporting systems, even though this requires extra time for such consideration.

## 6 HOW H2H CAPABILITIES SUPPORTED COVID‐19 RESPONSE EFFORTS

During the Covid‐19 pandemic, the H2H team's training and experience with managing patients with highly complex conditions enabled the team to rapidly pivot to supporting patients recovering from Covid‐19 in the community, especially elderly patients as well as middle‐aged patients with multiple disease conditions. The Covid‐19 situation required the H2H patient care team—both those based in the community as well as those stationed in the hospitals—to substantially increase the use of telehealth support for monitoring and virtual consultations. Going forward, we can already see that we will continue making use of telehealth for these purposes, though we also recognize the need to maintain direct “human touch” provided by face‐to‐face contact with our H2H patients in their homes and in their community. We are early in the process of experimenting with how to effectively combine telehealth with face‐to‐face engagement for postdischarge community support.

As Covid‐19 resulted in a sudden and prolonged surge in hospital admissions, there was an urgent need to provide more inpatient hospital beds. In April 2020, we already had nearly 3 years of experience with the H2H program, and we were able to ramp up our efforts in selected public hospitals and communities to reduce the risks of discharging current inpatients who fit the H2H program profile, or close to it, who did not have Covid‐19. In the hospitals where we were able to do this, this was a helpful measure during critical periods when it was necessary to cope with the sudden surge in demand for inpatient beds.

These are two good examples of how the H2H program made a specific contribution to the resiliency and capacity of our overall public healthcare system during the early phases of the Covid‐19 national response, and these were byproduct benefits of having the program in place.

## 7 MOVING FORWARD: BUILDING ON AI AND COMMUNITY CARE CAPABILITIES

Going forward, we can see that the core features of this type of program—using an AI prediction model in conjunction with clinical evaluation to support an evaluation process for selection in combination with postdischarge community care—will serve as useful foundational experience for dealing with the much wider range of population health challenges we are now facing and will continue to face in years to come. As part of the recently announced Healthier SG policy [16, 17], Singapore's Ministry of Health has already announced that it is transitioning the public healthcare sector funding scheme from a reimbursement‐for‐services‐provided approach to a capitation approach where each of the three public healthcare clusters will be assigned to cover a nonoverlapping part of the total population and receive a corresponding per‐capita‐based budget allotment. Hence, there will be a much stronger financial incentive for public sector healthcare providers to emphasize prevention and proactive population health measures to reduce disease occurrence rates and severity levels. Similarly, the three public healthcare clusters will have increasing incentive to continue with their own tailored version of H2H‐like efforts to reduce the duration of inpatient stays and reduce rates of multiple readmissions. To better respond to the increasing public healthcare load of Singapore's rapidly ageing population, our three major hospital clusters will have greater flexibility to allocate their resources to address both upstream population health measures as well as downstream efforts to mitigate the load of acute care needs.

As international public health studies have shown that people with a regular family physician are generally healthier and have fewer hospitalizations and emergency department visits, there is now a recently introduced accompanying national engagement effort to strongly encourage every resident—especially adults and the elderly—to establish a relationship with a family physician [18]. This is important as only three in five Singaporeans currently have a regular doctor [19].

Given this new approach for moving forward with Singapore's public sector healthcare, we anticipate there will be new efforts to use the existing sources of data already available for patient care support—in combination with new or additional sources of health and social data—to create new types of targeted prediction and recommendation models to support upstream community and population health efforts aimed at reducing the need for inpatient hospitalization and at shortening the duration of those stays. We also anticipate there will be new ways in which various aspects of the postdischarge community care model we have demonstrated with H2H—targeted at the subpopulation of people with the most complex needs—will be incorporated into a new generation of community care and population health efforts, and also expanded beyond only the elderly with the most complex care conditions to include broader segments of the resident population as well. The H2H effort also demonstrated the importance of complementary investment in an accompanying program dashboard for facilitating the tracking of outcomes and results and for making relevant comparisons.

In conclusion, the H2H program demonstrated that we can: (i) use an AI prediction model to support targeted ways of engaging with a specific segment of the population based on their public healthcare system interaction history and risk profile, (ii) use this as part of a multitier evaluation process to assess patient suitability for special programs, (iii) have multidisciplinary care teams develop personalized patient care plans, and (iv) do this on a national scale on a recurring production basis. As such, our overall assessment is that this national H2H effort, which started in 2017 and ran as a coordinated national program through 2022, has been very useful for providing experience and capabilities that are needed to meet the challenges of our next generation of public healthcare engagement.

## AUTHOR CONTRIBUTIONS

**John Abisheganaden**: Conceptualization (equal); data curation (equal); funding acquisition (lead); investigation (equal); methodology (supporting); project administration (equal); resources (equal); writing—original draft (equal); writing—review and editing (equal). **Kheng Hock Lee**: Conceptualization (equal); data curation (equal); funding acquisition (equal); investigation (equal); methodology (supporting); project administration (equal); resources (equal); writing—original draft (equal); writing—review and editing (equal). **Lian Leng Low**: Conceptualization (equal); data curation (equal); funding acquisition (equal); investigation (equal); methodology (supporting); project administration (equal); resources (equal); writing—original draft (equal); writing—review and editing (equal). **Eugene Shum**: Conceptualization (equal); data curation (equal); funding acquisition (equal); investigation (equal); methodology (supporting); project administration (equal); resources (equal); writing—original draft (equal); writing—review and editing (equal). **Han Leong Goh**: Conceptualization (equal); data curation (lead); formal analysis (equal); investigation (equal); methodology (equal); resources (equal); software (equal); writing—original draft (equal); writing—review and editing (equal). **Christine Gia Lee Ang**: Conceptualization (equal); data curation (equal); formal analysis (equal); funding acquisition (equal); investigation (equal); methodology (equal); project administration (equal); resources (equal); software (equal); writing—original draft (supporting); writing—review and editing (supporting). **Andy Wee An Ta**: Conceptualization (equal); data curation (equal); formal analysis (lead); funding acquisition (equal); investigation (equal); methodology (lead); project administration (equal); resources (equal); software (lead); writing—original draft (supporting); writing—review and editing (equal). **Steven M. Miller**: Writing—original draft (lead); writing—review and editing (lead).

## CONFLICT OF INTEREST STATEMENT

The authors declare there are no conflict of interest.

## ETHICS STATEMENT

This article is a practice‐oriented case study that drew upon the program experience and professional knowledge of the co‐authors. As such, the effort for this particular article did not involve human participation in research study, and IRB review was not required.

## INFORMED CONSENT

Informed consent was not required.

## Data Availability

Data sharing is not applicable to this article as no new data were created or analyzed in this study.

## Footnotes

* Descriptions of two Singapore precursor efforts to the postdischarge community support aspect of Singapore's Hospital‐to‐Home program are given in [3] and [4].

^†^
Other Ministry of Health data indicates that admissions for public sector acute hospitals for 2021 and 2022 were 459,927 and 480,041, respectively.

^‡^
The formula used for each measure is as follows: Net Change in Bed Days = Change in Bed Days of Enrolled Patients – Average Change in Bed Days of Nonenrolled Patients of the same CCI score (control group); where Change in Bed Days = Cumulative Bed Days 180 days post the Index Admission – Cumulative Bed Days 180 days prior to the index admission. Similarly for Net Change in visits to Accident and Emergency unit.

^§^
Reference [12] is an example of rigorously designed and executed randomized control trial study of providing a cohort of “superutilizers”—patients with very high use of healthcare services—with an intervention that is similar in concept to the postdischarge community support and care aspect of our H2H program. This study found that readmission rates were not lower among patients randomly assigned to the intervention program than among those who received usual care. The authors state their results suggest that there are challenges for superutilizer programs aimed at medically and socially complex populations.
